# Dual-input spatio-temporal transformer model: Predicting the efficacy of NACT in breast cancer based on DCE-MRI images

**DOI:** 10.1016/j.isci.2025.114433

**Published:** 2025-12-13

**Authors:** Hongbo Song, Guanliang Dong, Yourong Chen, Zhangquan Wang, Liyuan Liu, Haidong Cui

**Affiliations:** 1 College of Information Science and Technology, Zhejiang Shuren University, Hangzhou, China; 2 Department of Decision & System Sciences, Saint Joseph’s University, Philadelphia, PA, USA; 3 Department of Breast Surgery, First Affiliated Hospital, Zhejiang University School of Medicine, Hangzhou, China

**Keywords:** Radiology, Oncology

## Abstract

Breast cancer remains a leading malignancy among women worldwide. Neoadjuvant chemotherapy (NACT) can shrink tumors and increase the possibility of breast-conserving surgery, yet many patients respond poorly, causing treatment delays and unnecessary toxicity. Accurate early prediction of NACT response is critical for tailoring therapy. To address this challenge, we developed a dual-input spatiotemporal transformer (DIST) model that analyzes dynamic contrast-enhanced MRI scans obtained before and after the first chemotherapy cycle. By jointly learning spatial and temporal tumor changes, DIST provides early, interpretable imaging-based predictions of treatment response. In validation, the model achieved high accuracy and strong generalizability across institutional and external datasets. These findings demonstrate that DIST enables reliable early assessment of chemotherapy efficacy, offering clinicians a valuable tool to optimize treatment strategies, minimize ineffective therapy, and improve patient outcomes.

## Highlights

• EiT2T enhances multi-scale features and local-global attention for lesion detail
• ST integrates spatial-temporal cues to better capture lesion evolution
• AFFC fuses multi-timepoint features and differences to improve classification

## Graphical abstract

**Figure:**
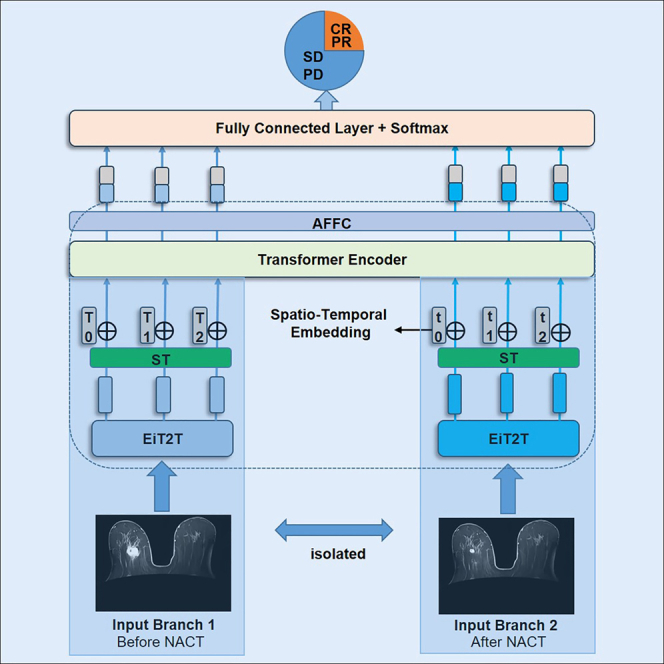

## Introduction

Breast cancer is the most prevalent malignant tumor among women globally, posing a significant health threat and impacting their survival.^1^^,^^2^ According to the global cancer statistics released by the International Agency for Research on Cancer of the World Health Organization, there were 19.97 million new cancer cases worldwide in 2022. Of these, breast cancer constituted over 2.3 million cases and resulted in 670,000 deaths, making it the cancer with the highest incidence and mortality among women globally. These statistics indicate that breast cancer continues to be a major health threat to women.^3^ Among the various treatment methods, neoadjuvant chemotherapy (NACT) is a particularly crucial approach. NACT reduces tumor size before surgery, making initially inoperable tumors resectable. It also significantly enhances the likelihood of breast conservation, which is essential for preserving the quality of life for patients4^4^^,^^5^ Additionally, NACT offers crucial insights into the tumor’s early response to chemotherapy drugs, helping physicians in developing precise treatment strategies.^6^

Despite its evident advantages, NACT is not effective for all patients and involves a relatively lengthy treatment cycle, typically encompassing 6 to 8 courses. Some patients exhibit poor responses to NACT, not achieving the intended therapeutic outcomes and potentially facing surgery delays, risking the loss of the optimal treatment window.^7^ In clinical practice, the assessment of therapeutic response after the first chemotherapy cycle provides a critical window for the early prediction of NACT efficacy. Numerous clinical studies have demonstrated that early imaging biomarkers derived from post-first-cycle imaging can reliably reflect tumor chemosensitivity and serve as prognostic indicators of the final treatment outcome.^8^^,^^9^^,^^10^ Early prediction at this timepoint enables clinicians to promptly identify non-responders and adjust treatment strategies, thereby improving patient prognosis and avoiding unnecessary adverse effects. Therefore, accurately predicting the efficacy of NACT at an early stage is essential for implementing personalized treatment plans, optimizing patient prognosis, and avoiding unnecessary treatments.

Evaluating the efficacy of NACT primarily involves clinical and pathological assessments. Clinical evaluation is based on the RECIST criteria, categorizing treatment responses into four groups—complete response (CR), partial response (PR), stable disease (SD), and progressive disease (PD)—by comparing changes in tumor diameter and volume before and after treatment.^11^^,^^12^ Studies have shown that patients who achieve CR or PR have better prognoses, especially those who achieve CR.^13^ Pathological evaluations employ the Miller-Payne and Residual Cancer Burden (RCB) systems to classify chemotherapy effects as either pathological complete response (pCR) or not pCR. This study will concentrate on predicting efficacy based on clinical response evaluations.

Medical imaging techniques such as mammography, computed tomography (CT), ultrasound (US), and magnetic resonance imaging (MRI) are crucial in diagnosing and assessing breast cancer. As a result, research aimed at predicting the effectiveness of NACT using medical imaging has attracted considerable interest in the medical community, with extensive literature available for refs.^14^^,^^15^^,^^16^^,^^17^ Among these imaging techniques, mammography struggles to differentiate blurred tumor margins, CT is limited in soft tissue contrast, and US is less effective in deep tissue assessment and detecting small lesions. These limitations affect the accuracy of NACT efficacy predictions for breast tumors. In contrast, breast MRI, particularly dynamic contrast-enhanced MRI (DCE-MRI), provides more detailed and comprehensive imaging by tracking contrast agent distribution within the body.^18^ Therefore, MRI-based methods for predicting NACT response hold greater potential for improving predictive accuracy. Current NACT prediction methods mainly utilize radiomics, which involves extracting numerous features from medical images to analyze disease patterns. Early studies primarily relied on manual feature extraction. For instance, Alina et al.^19^ quantified DCE-MRI data to predict NACT response, while Aghaei et al.^20^ used computer-aided techniques to analyze MRI features for chemotherapy outcome predictions. However, these manual approaches are limited in efficiency.

On the other hand, artificial intelligence has been increasingly integrated into clinical oncology for decision support and precision medicine in recent years. In particular, Medical Explainable Artificial Intelligence (MEAI) systems have shown great potential in assisting radiologists and oncologists by providing interpretable predictions for treatment planning and response assessment. In the context of NACT, MEAI-based approaches can help clinicians identify non-responders at an early stage, optimize therapeutic strategies, and avoid unnecessary toxicity or delays in surgery. Several studies have demonstrated that AI-driven imaging biomarkers derived from MRI can serve as valuable adjuncts to conventional clinical evaluation, supporting individualized treatment decisions and improving patient outcomes. Therefore, developing an interpretable and clinically applicable model for NACT efficacy prediction is of both scientific and translational importance.

For the NACT prediction, Ha et al.^21^ applied convolutional neural networks (CNNs) to extract imaging features from pre-treatment MRI and successfully predicted breast cancer patient responses to NACT, achieving high overall accuracy. Expanding on MRI images, Peng et al.^22^ incorporated molecular information related to breast tumors to predict NACT efficacy, while Joo et al.^23^ developed a novel branch CNN that combined MRI images with genomic data to improve NACT response predictions. However, the above models primarily considered pre-chemotherapy imaging data and did not account for dynamic changes during or post-treatment, which limits their ability to comprehensively and accurately reflect actual tumor response. To address this, Qu et al.^24^ proposed a CNN model based on pre- and post-NACT MRI images to predict NACT efficacy, and Kavya et al.^25^ utilized a CNN on pre- and post-NACT DCE-MRI images for response evaluation. Results indicated that models incorporating both pre- and post-NACT imaging data showed superior predictive accuracy compared to single-time-point imaging. Furthermore, Li et al.^26^ combined pre- and post-chemotherapy MRI images with clinicopathological data to develop a novel prediction model, significantly enhancing the accuracy of chemotherapy response predictions.

It is worth noting that the quality and imaging parameters of DCE-MRI images can be influenced by various factors, such as the timing of contrast agent injection, minor changes in patient positioning, and technical discrepancies between different scanners. These factors can introduce visual and quantitative differences in images acquired before and after chemotherapy. Several studies have employed independent CNNs to process images from specific time points, with each CNN operating independently and without shared information.^24^^,^^25^^,^^26^ This approach restricts the model’s ability to integrate information across different time points, limiting the deep networks' potential to fully capture and learn from temporal changes. Consequently, effectively mapping subtle differences and potential inconsistencies across time points to predict NACT outcomes remains challenging, prompting researchers to explore novel model architectures.

The transformer, a deep neural network architecture based on a multi-head self-attention mechanism, was initially designed for sequence-to-sequence tasks in natural language processing. The vision transformer (ViT) was the first successful application of the Transformer model to image classification tasks.^27^ ViT has achieved state-of-the-art performance on multiple benchmarks, demonstrating its powerful capability for in-depth image analysis. Compared to CNNs, ViT’s primary advantage lies in its ability to capture global relationships across various time points in images, facilitating the integration of multi-timepoint imaging information and enhancing the model’s predictive capability for whole image sequences. Li et al.^28^ implemented a Transformer-based attention mechanism to identify correlations between MRI image features and genomic data for predicting NACT outcomes. Similarly, Tong et al.^29^ proposed a dual-input Transformer model, pioneering the use of ViT for multi-timepoint US image-based NACT outcome prediction, achieving promising results. However, Tong et al. employed only two US images (pre- and post-treatment), whereas DCE-MRI involves multiple images in both pre- and post-treatment series, requiring more advanced analytical methods to capture dynamic changes across images.

Overall, conventional ViT models still face certain limitations in handling such complex multi-timepoint images. First, in traditional ViT, images are typically partitioned into fixed-size patches, each treated as a “token” input to the Transformer, potentially leading to a loss of local detail, especially in tasks demanding fine image detail. Second, standard ViT employs static positional embeddings, using the same fixed position matrix for all images, without a dedicated temporal embedding module, thus limiting its ability to capture dynamic changes across different time points. Lastly, ViT relies on a CLS token for classification decisions, which aggregates information less efficiently for time-sequential tasks. Consequently, enhancing ViT to more effectively process DCE-MRI images and fully utilize multi-timepoint data remains an ongoing challenge. To the best of the authors' knowledge, no study has yet employed a branched ViT model with DCE-MRI images for predicting NACT efficacy.

In this study, we introduce a Dual-Input spatiotemporal Transformer (DIST) model tailored to analyze pre- and post-NACT DCE-MRI images to predict chemotherapy responses in patients with breast cancer. Although radiologists can visually assess post-treatment images, our model offers an objective, reproducible, and standardized evaluation approach that reduces inter-observer variability caused by subjective judgment. Moreover, it enables the extraction of subtle radiomic and spatiotemporal features from DCE-MRI that often exceed the perceptual limits of the human eye and may reflect treatment response at an earlier stage. The DIST model is optimized to address subtle discrepancies and potential inconsistencies across images from different time points. Specifically, pre- and post-NACT images are processed individually to create separate input sequences.

The model’s performance improvements are achieved through the integration of three key components: an Enhanced isolated Tokens-to-Token Patch Embedding Module (EiT2T), a spatiotemporal Embedding Module (ST), and an Adaptive Feature Fusion and Classification Embedding Module (AFFC). First, we introduce the EiT2T module, an innovation based on the conventional Tokens-to-Token (T2T) technique.^30^ Although T2T partially addresses the limitations of tokenization in traditional Transformer models, it generally uses fixed patch sizes. By incorporating multi-scale feature extraction, the EiT2T module enables the model to capture a broader range of features, thereby enhancing sensitivity to subtle details. Additionally, a local-global attention mechanism further balances local and global information capture, enhancing the model’s capacity to detect intricate lesion features and improving complex lesion recognition accuracy. Next, we developed the ST module, which replaces traditional static positional embeddings. Conventional positional embeddings use a fixed position matrix shared across images, disregarding differences among individual images. By adopting dynamic positional embeddings and integrating temporal embeddings, this module enables comprehensive embedding of both spatial and temporal information. The generation of unique dynamic embedding vectors for each image allows the model to more accurately reflect changes across spatial and temporal dimensions. Finally, to address the limitations of the CLS token in aggregating information for classification decisions, we introduced the AFFC module. By adaptively fusing image features from different time points and emphasizing feature differences crucial to classification, AFFC significantly enhances the model’s discriminative capability. We validated the effectiveness of the DIST model on a private breast MRI dataset and compared its performance against other existing models. The main contributions of this work are as follows.

(1) Enhanced isolated Tokens-to-Token (EiT2T) with Multi-Scale Features and Local-Global Attention Mechanism: While traditional T2T modules preserve local structural information by progressively decomposing image patches, they fall short in capturing multi-scale features and global context. Building on this approach, we propose the EiT2T module, which integrates multi-scale convolution and local-global attention mechanisms. EiT2T effectively preserves edges, textures, and other structural details within the images, significantly enhancing the model’s ability to capture lesion characteristics in complex medical images.
(2) Spatiotemporal Embedding Module (ST) Integrating Spatial and Temporal Information: To address the limitation of traditional static positional embeddings in capturing temporal information, we designed the ST module, which combines dynamic positional embeddings with temporal embeddings, embedding them directly into each encoded token. The ST module strengthens the model’s capacity to integrate spatial and temporal information, effectively utilizing inter-image temporal sequences to more accurately capture and analyze pathological changes over time, thereby improving the model’s understanding of image evolution.
(3) Adaptive Feature Fusion and Difference Modeling-Based Classification Method: We developed a novel classification method, Adaptive Feature Fusion and Classification (AFFC), which employs an adaptive weighting mechanism to fuse features extracted from different time points (pre- and post-chemotherapy) and directly models their feature differences. These results are integrated into the classification embedding, significantly enhancing the model’s discriminative power.

## Results

In this section, we provide a detailed description of a series of experiments conducted to comprehensively evaluate the performance of the DIST model in predicting NACT efficacy. The experimental design follows rigorous scientific protocols, covering the specifics of the experimental setup, comparisons with current state-of-the-art (SOTA) methods, and an in-depth analysis of the contributions of various model components.

### Data description

#### Data size and distribution

This retrospective study included 181 patients with breast cancer who received treatment at the First Affiliated Hospital of Zhejiang University School of Medicine between 2021 and 2023. Inclusion criteria were as follows: (1) histopathologically confirmed breast cancer; (2) receipt of neoadjuvant chemotherapy (NACT); (3) availability of complete pre- and post-treatment MRI data. Exclusion criteria included: (1) patients who did not receive NACT; (2) incomplete MRI data. According to these criteria, 9 patients were excluded due to incomplete MRI imaging, and 172 patients were finally included in the study.

Therapeutic response labels were recorded based on clinical efficacy evaluations. Patients achieving complete response (CR) or partial response (PR) were categorized as responders (positive class, label = 1), while those with stable disease (SD) or progressive disease (PD) were defined as non-responders (negative class, label = 0). The dataset was divided into training (*n* = 103), validation (*n* = 34), and testing (*n* = 35) sets at a ratio of 3:1:1. In the original dataset, response labels were imbalanced (CR = 20, PR = 129, SD = 23), resulting in 149 responders (positive) and 23 non-responders (negative), with a ratio of approximately 6.5:1.

Such an imbalance could cause the model to bias toward the majority class, limiting its ability to learn minority-class features. To address this issue, multiple data augmentation techniques were applied to the training set, including horizontal flipping, vertical flipping, and fixed-angle rotations (±15° and ±30°). After augmentation, the total number of training samples increased from 103 to approximately 515, including about 260 positive and 255 negative cases, achieving an approximately 1:1 balance. This augmentation strategy effectively enhanced the model’s ability to learn minority-class features and improved its generalization performance. The validation and testing sets were not augmented, maintaining their original distributions (positive: negative ≈6.5:1) to ensure objective model evaluation and clinical generalizability.

#### Dynamic contrast-enhanced-magnetic resonance imaging acquisition parameters

All patients underwent DCE-MRI before and after NACT using a 3.0-T MRI scanner equipped with a dedicated 16-channel breast coil. A fat-suppressed T1-weighted three-dimensional fast dynamic contrast-enhanced sequence (3D-VIBE) was applied. The key parameters were as follows: repetition time = 4.5 ms, echo time = 1.8 ms, flip angle = 12°, slice thickness = 2.0 mm, interslice gap = 0.5 mm, field of view (FOV) = 360 mm × 360 mm, matrix = 320 × 320.

The contrast agent gadobutrol (0.1 mmol/kg) was injected intravenously through the antecubital vein at a rate of 2.5 mL/s, followed by a 20 mL saline flush. Dynamic scanning commenced immediately after contrast injection, with a temporal resolution of 8 s per frame and a total of 40 frames acquired over approximately 6 min. The imaging coverage included both breasts and the axillary regions. Post-processing automatically generated time-signal intensity curves for the analysis of early and delayed enhancement characteristics of the lesions.

#### Clinical characteristics and chemotherapy regimens

The 172 included patients were 29–68 years old, with a median age of 47 years. According to immunohistochemistry, molecular subtypes were distributed as follows: Luminal A (*n* = 56, 32.6%), Luminal B (*n* = 62, 36.0%), HER2-enriched (*n* = 28, 16.3%), and triple-negative breast cancer (TNBC, *n* = 26, 15.1%).

All patients received standardized NACT regimens, including the EC-T regimen (epirubicin + cyclophosphamide followed by paclitaxel, *n* = 93, 54.1%), the TCbH regimen (docetaxel + carboplatin + trastuzumab ± pertuzumab, *n* = 41, 23.8%), and other individualized regimens based on taxane- or anthracycline-containing combinations (*n* = 38, 22.1%). Each patient underwent pre- and post-NACT DCE-MRI examinations, and treatment response was assessed according to pathological findings (CR/PR vs. SD/PD).

In this study, patients received standardized NACT regimens, primarily EC-T (epirubicin + cyclophosphamide followed by paclitaxel) and TCbH (docetaxel + carboplatin + trastuzumab ± pertuzumab), corresponding to HER2-negative and HER2-positive subtypes, respectively. All treatments followed NCCN guidelines. The proposed model was designed to predict overall clinical response (CR/PR vs. SD/PD) across different NACT regimens rather than regimen-specific outcomes.

#### Image preprocessing and data construction

Image preprocessing was a crucial step to ensure feature stability and model performance. DCE-MRI data were collected, each including scans at two time points: before NACT and after the first chemotherapy cycle, with six dynamic phases (S0–S5) acquired at each time point.

**Figure 1:**
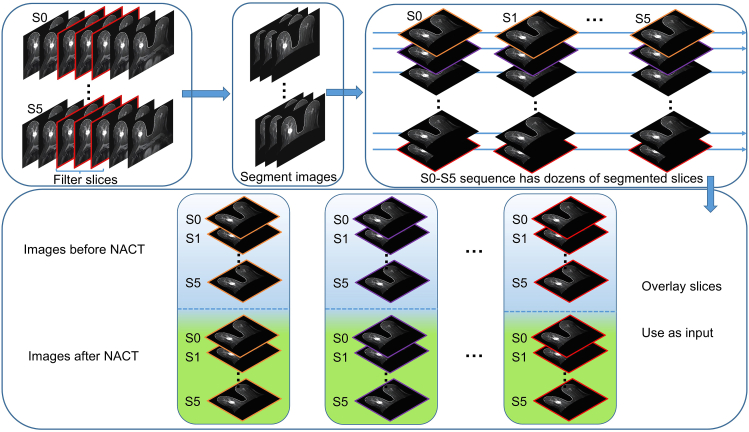
Flowchart of image data processing The figure illustrates the DCE-MRI preprocessing workflow. Slices from each dynamic phase (S0–S5) were filtered and segmented to retain breast tissue while removing the skin and thoracic wall. Corresponding slices across the six phases were overlaid and stacked into six-channel composite images. Each patient contributed pre- and post-NACT image sets for model training. NACT, neoadjuvant chemotherapy.

As illustrated in Figure 1, breast region segmentation was performed to remove the skin and thoracic wall, retaining only the breast tissue for analysis. In the S1 phase, the slice showing the largest tumor diameter was selected as the central reference slice, and continuous slices within approximately ±2 cm were extracted to cover the entire tumor region. To minimize spatial misalignment between pre- and post-treatment scans, rigid alignment was applied to the selected slices to improve temporal consistency.

All MR scans followed a unified acquisition protocol, with fixed contrast-injection timing and six pre-defined dynamic time points, ensuring consistency along the temporal dimension. For model construction, corresponding segmented slices from S0–S5 were extracted and stacked as six-channel composite images, producing multiple six-channel image sets per patient at both pre- and post-NACT time points for model training and analysis.

### Comparison with baseline methods

To comprehensively assess the performance of the DIST model in predicting the efficacy of NACT for breast cancer, we conducted an extensive comparison on our breast DCE-MRI dataset with several SOTA models. This comparison included VGG19, MobileNetV2, ResNet50, DenseNet169, ViT, and two specialized models proposed for similar tasks: the Siamese CNN by Byra et al.^31^ and the DiT model by Tong et al.^29^ Quantitative results are summarized in Tables 1 and 2, demonstrating the performance advantages of the DIST model over these established algorithms.

**Table 1:** Model performance test results on the validation set based on a breast DCE-MRI dataset

Model	AUC(95% CI)	Accuracy(95% CI)	Specificity(95% CI)	Sensitivity(95% CI)
VGG19	0.757 (0.725, 0.789)	0.739 (0.710, 0.768)	0.719 (0.680, 0.758)	0.712 (0.678, 0.746)
MobileNetV2	0.761 (0.730, 0.792)	0.744 (0.715, 0.773)	0.723 (0.685, 0.761)	0.726 (0.692, 0.760)
ResNet50	0.768 (0.736, 0.800)	0.753 (0.724, 0.782)	0.727 (0.689, 0.765)	0.735 (0.700, 0.770)
DenseNet169	0.780 (0.750, 0.810)	0.760 (0.731, 0.789)	0.731 (0.692, 0.770)	0.748 (0.714, 0.782)
ViT	0.804 (0.774, 0.834)	0.772 (0.743, 0.801)	0.750 (0.712, 0.788)	0.762 (0.728, 0.796)
Siamese CNN	0.834 (0.805, 0.863)	0.809 (0.781, 0.837)	0.800 (0.768, 0.832)	0.795 (0.762, 0.828)
DiT	0.883 (0.857, 0.909)	0.840 (0.812, 0.868)	0.824 (0.792, 0.856)	0.820 (0.787, 0.853)
Our model	**0.924 (0.901, 0.947)**	**0.869 (0.844, 0.894)**	**0.847 (0.818, 0.876)**	**0.843 (0.814, 0.872)**

**Table 2:** Model performance test results on the test set based on a breast DCE-MRI dataset

Model	AUC(95% CI)	Accuracy(95% CI)	Specificity(95% CI)	Sensitivity(95% CI)
VGG19	0.745 (0.713, 0.777)	0.730 (0.701, 0.759)	0.706 (0.670, 0.742)	0.700 (0.664, 0.736)
MobileNetV2	0.752 (0.721, 0.783)	0.738 (0.709, 0.767)	0.712 (0.675, 0.749)	0.720 (0.685, 0.755)
ResNet50	0.768 (0.726, 0.800)	0.741 (0.712, 0.770)	0.718 (0.682, 0.754)	0.727 (0.692, 0.762)
DenseNet169	0.782 (0.752, 0.812)	0.749 (0.720, 0.778)	0.725 (0.689, 0.761)	0.739 (0.704, 0.774)
ViT	0.782 (0.752, 0.812)	0.758 (0.729, 0.787)	0.741 (0.705, 0.777)	0.753 (0.718, 0.788)
Siamese CNN	0.824 (0.796, 0.852)	0.805 (0.778, 0.832)	0.792 (0.758, 0.826)	0.788 (0.753, 0.823)
DiT	0.876 (0.851, 0.901)	0.831 (0.804, 0.858)	0.813 (0.779, 0.847)	0.811 (0.776, 0.846)
Our model	**0.913 (0.891, 0.935)**	**0.858 (0.832, 0.884)**	**0.840 (0.808, 0.872)**	**0.835 (0.802, 0.868)**

**Figure 2:**
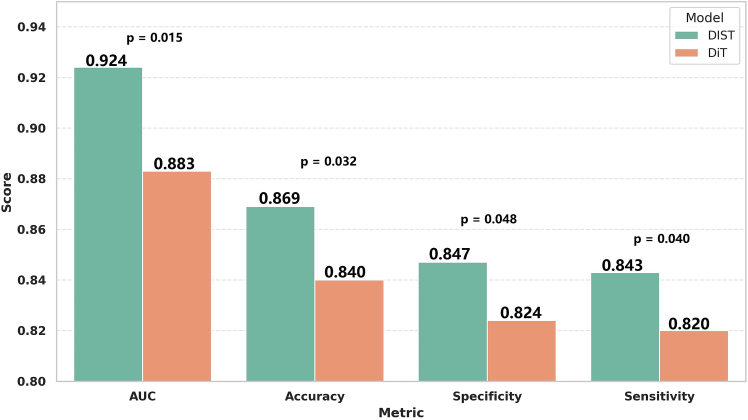
Performance comparison on validation set: DIST vs. DiT with *p*-values Bars represent the mean ± 95% CI across the 5-fold cross-validation. Statistical significance was assessed using the DeLong test for AUC, the McNemar test for accuracy, and paired t-tests for specificity and sensitivity. The DIST model demonstrated significantly higher AUC, accuracy, specificity, and sensitivity than the DiT model (*p* < 0.05).

The tabulated results highlight the superior performance of the proposed DIST model in predicting the efficacy of neoadjuvant chemotherapy for breast cancer, with statistically significant improvements. As shown in Table 1 and Figure 2, DIST achieved an AUC of 0.924 on the validation set, outperforming the closest competitor, the DiT model (AUC = 0.883), by 4.64%. Statistical analysis using the DeLong test confirmed the significance of this improvement (*p* = 0.015), indicating that the performance gain is unlikely due to random variation. The higher AUC suggests a stronger discriminative ability of the model in distinguishing responders from non-responders, underscoring its clinical applicability.

**Figure 3:**
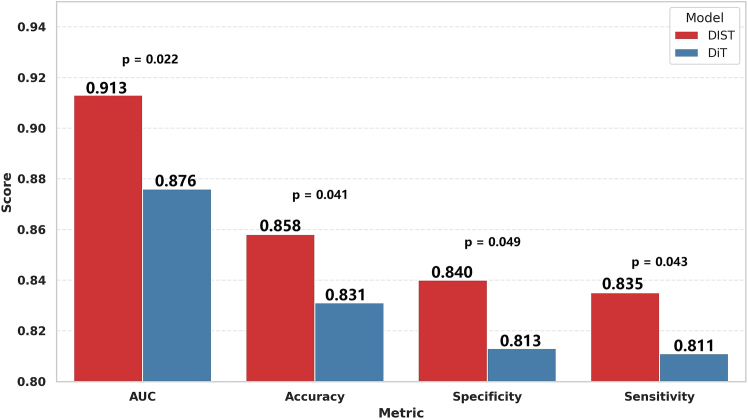
Performance comparison on test set: DIST vs. DiT with *p*-values Bars represent the mean ± 95% CI across the 5-fold cross-validation. Statistical significance was assessed using the DeLong test for AUC, the McNemar test for accuracy, and paired t-tests for specificity and sensitivity. The DIST model demonstrated significantly higher AUC, accuracy, specificity, and sensitivity than the DiT model (*p* < 0.05).

On the test set, as shown in Table 2 and Figure 3, DIST maintained its advantage with an AUC of 0.913, surpassing DiT (AUC = 0.876) by 4.22%, with the DeLong test again confirming statistical significance (*p* = 0.022), further demonstrating the robustness of the model across data subsets. In terms of accuracy, DIST achieved 0.869 on the validation set, higher than DiT’s 0.840. A McNemar test showed this difference to be statistically significant (*p* = 0.032). A similar advantage was observed on the test set (0.858 vs. 0.831; *p* = 0.041), indicating the superior reliability of DIST in actual classification tasks. Furthermore, DIST exhibited notable improvements in two key clinical metrics—specificity and sensitivity—both critical for patient prognosis. On the validation set, DIST achieved a specificity of 0.847 and a sensitivity of 0.843, outperforming DiT (0.824 and 0.820) by 2.79% and 2.3%, respectively. These improvements remained consistent across the 5-fold cross-validation (*p* = 0.048 for specificity, *p* = 0.040 for sensitivity). On the test set, DIST achieved a specificity of 0.840 and a sensitivity of 0.835, continuing to outperform DiT and demonstrating its dual advantage in reducing both false positives and false negatives.

Overall, all reported *p*-values were below 0.05, confirming that the performance improvements of DIST over DiT across AUC, accuracy, specificity, and sensitivity are statistically significant. This consistent statistical evidence underscores the robustness and reliability of the DIST model’s superiority.

To further evaluate the generalizability of the proposed model, we conducted transfer learning experiments on the publicly available TCIA I-SPY2 dataset. Although a small subset of the I-SPY2 dataset was utilized during model pretraining to enhance feature initialization, the training and external validation subsets were completely independent, with no patient overlap between them. The number of I-SPY2 samples used for pretraining was minimal and does not affect the statistical independence of the external evaluation. In addition, the I-SPY2 dataset itself is a large-scale, multi-center public cohort containing 985 patients, which provides a sufficiently diverse and representative population for robust external validation. We also plan to further evaluate the proposed DIST model on the DUKE breast cancer dataset and other multi-center cohorts in future work to strengthen its external validation and clinical generalizability. Specifically, the model was first pre-trained on our in-house dataset, after which the learned weights were fine-tuned on I-SPY2 to accommodate its imaging characteristics and annotation patterns. To better approximate real-world clinical scenarios, only a subset of clearly labeled and high-quality breast MRI samples from the I-SPY2 dataset was selected for training. The backbone of the model architecture was kept unchanged during fine-tuning. For a fair comparison, all baseline models (including ViT, DiT, and Siamese CNN) underwent the same transfer learning procedure under identical data splits and training settings.

As summarized in the Table 3, the proposed model maintained superior performance on the I-SPY2 validation set after fine-tuning, achieving an AUC of 0.886 (95% CI: 0.860,0.912), an accuracy of 0.832 (95% CI: 0.807,0.857), a specificity of 0.819 (95% CI: 0.786,0.852), and a sensitivity of 0.825 (95% CI: 0.793,0.857). These results consistently outperformed all baseline methods, including ViT (AUC: 0.756) and DiT (AUC: 0.854). This demonstrates that our model not only performs well on internal data but also exhibits strong robustness and generalizability when transferred to an external public dataset, highlighting its potential for clinical deployment across institutions. The DIST model demonstrated significant benefits over other advanced models across all major performance metrics. These results not only validate the efficiency of the DIST model but also indicate its ability to provide more accurate and reliable predictions when handling complex neoadjuvant chemotherapy data for breast cancer.

**Table 3:** Model performance comparison on the I-SPY2 public dataset

Model	AUC(95% CI)	Accuracy(95% CI)	Specificity(95% CI)	Sensitivity(95% CI)
VGG19	0.722 (0.693, 0.751)	0.709 (0.683, 0.735)	0.693 (0.659, 0.727)	0.702 (0.671, 0.733)
MobileNetV2	0.728 (0.699, 0.757)	0.717 (0.691, 0.743)	0.699 (0.666, 0.732)	0.715 (0.685, 0.745)
ResNet50	0.735 (0.706, 0.764)	0.723 (0.697, 0.749)	0.703 (0.670, 0.736)	0.713 (0.683, 0.743)
DenseNet169	0.741 (0.712, 0.770)	0.728 (0.702, 0.754)	0.710 (0.677, 0.743)	0.721 (0.690, 0.752)
ViT	0.756 (0.726, 0.786)	0.741 (0.715, 0.767)	0.730 (0.696, 0.764)	0.736 (0.705, 0.767)
Siamese CNN	0.801 (0.773, 0.829)	0.782 (0.756, 0.808)	0.773 (0.740, 0.806)	0.778 (0.747, 0.809)
DiT	0.854 (0.828, 0.880)	0.809 (0.784, 0.834)	0.793 (0.759, 0.827)	0.803 (0.771, 0.835)
Our model	**0.886 (0.860, 0.912)**	**0.832 (0.807, 0.857)**	**0.819 (0.786, 0.852)**	**0.825 (0.793, 0.857)**

### Comparison of different chemotherapy time points

To further investigate the performance of the NACT response prediction model at different imaging time points, we designed a series of experiments using three data configurations: pre-chemotherapy imaging only, post-first-cycle chemotherapy imaging only, and a fusion of both time points. By conducting comparative analyses on both the validation and test sets, we aimed to evaluate the impact of temporal imaging strategies on predictive performance. The corresponding results are summarized in Tables 4 and 5.

**Table 4:** Model performance test results on the validation set based on different chemotherapy time points

Model	Time	AUC(95% CI)	Accuracy(95% CI)	Specificity(95% CI)	Sensitivity(95% CI)
T2T-ViT	Pre	0.623 (0.582, 0.664)	0.600 (0.565, 0.635)	0.546 (0.503, 0.589)	0.569 (0.530, 0.608)
T2T-ViT	Post	0.846 (0.820, 0.872)	0.818 (0.790, 0.846)	0.810 (0.778, 0.842)	0.804 (0.771, 0.837)
Our model	**Pre and Post**	**0.924 (0.901, 0.947)**	**0.869 (0.844, 0.897)**	**0.847 (0.818, 0.876)**	**0.843 (0.814, 0.872)**

**Table 5:** Model performance test results on the test set based on different chemotherapy time points

Model	Time	AUC(95% CI)	Accuracy(95% CI)	Specificity(95% CI)	Sensitivity(95% CI)
T2T-ViT	Pre	0.617 (0.576, 0.658)	0.589 (0.554, 0.624)	0.512 (0.469, 0.555)	0.525 (0.486, 0.564)
T2T-ViT	Post	0.827 (0.799, 0.855)	0.812 (0.784, 0.840)	0.803 (0.770, 0.836)	0.794 (0.761, 0.827)
Our model	**Pre and Post**	**0.913 (0.891, 0.935)**	**0.858 (0.832, 0.884)**	**0.840 (0.808, 0.872)**	**0.835 (0.802, 0.868)**

**Figure 4:**
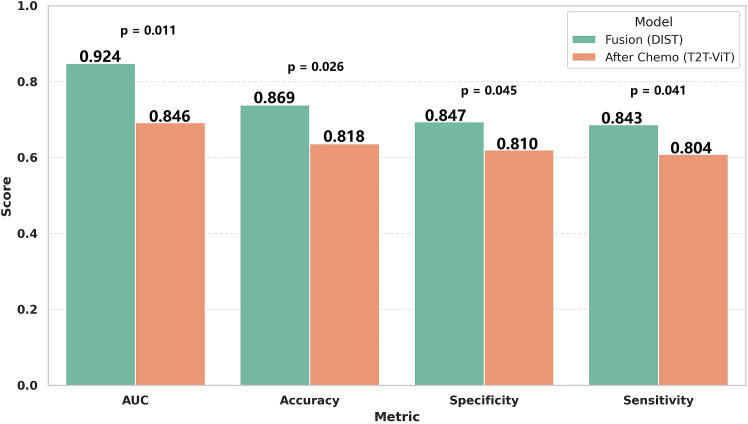
Validation set: fusion vs. after chemo (T2T-ViT) Bars represent the mean ± 95% CI across the 5-fold cross-validation. Statistical significance was evaluated using the DeLong test for AUC, the McNemar test for accuracy, and paired t-tests for specificity and sensitivity. The fusion model demonstrated significantly improved AUC, accuracy, specificity, and sensitivity over the single-time-point model (*p* < 0.05).

As shown in Table 4 and Figure 4, the T2T-ViT model based on pre-chemotherapy images alone performed poorly on the validation set, with an AUC of only 0.623 and an accuracy of 0.600, indicating prediction performance close to random guessing (AUC = 0.5). In contrast, when using post-first-cycle chemotherapy images alone, the model’s performance improved substantially, achieving an AUC of 0.846 and an accuracy of 0.818—an increase of 35.8% and 36.3%, respectively. This significant improvement suggests that post-chemotherapy images contain more informative structural and functional cues related to treatment response. In comparison, our proposed model, which leverages the fusion of both pre- and post-chemotherapy images, significantly outperformed single time point inputs. The model achieved an AUC of 0.924, representing a 9.2% improvement over the post-chemotherapy-only input (DeLong test, *p* = 0.011), and an accuracy of 0.869, representing a 6.2% improvement (McNemar test, *p* = 0.026). In terms of specificity and sensitivity, the model achieved 0.847 and 0.843, respectively—improvements of 2.8% and 2.3% over the post-chemotherapy model, both statistically significant (*p* < 0.05). These findings indicate that combining multi-temporal imaging enables the model to more comprehensively capture biological changes before and after treatment, thereby improving both predictive accuracy and stability.

**Figure 5:**
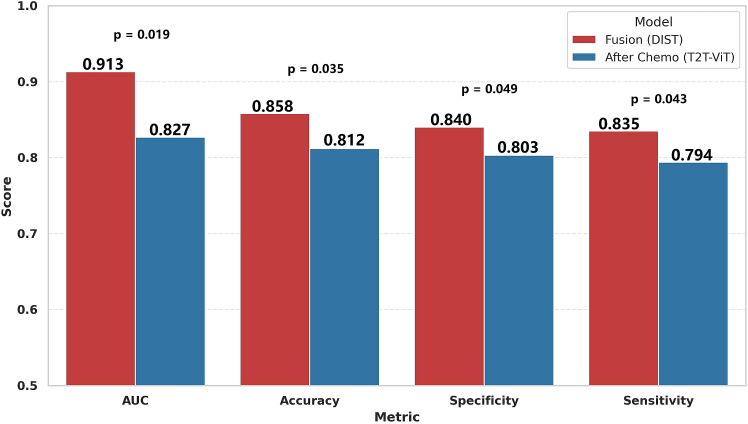
Test set: fusion vs. after chemo (T2T-ViT) Bars represent the mean ± 95% CI across the 5-fold cross-validation. Statistical significance was assessed using the DeLong test for AUC, the McNemar test for accuracy, and paired t-tests for specificity and sensitivity. The Fusion model achieved significantly higher AUC, accuracy, specificity, and sensitivity than the single-time-point model (*p* < 0.05).

The results on the test set (Table 5; Figure 5) further support the observations from the validation set. The T2T-ViT model achieved an AUC of 0.617 using pre-chemotherapy images, which increased to 0.827 when using post-chemotherapy images—a 34.0% improvement, again highlighting the advantages of post-treatment imaging. However, our DIST model with fused imaging inputs performed even better, achieving an AUC of 0.913, which is 8.6% higher than the post-chemotherapy-only input (DeLong test, *p* = 0.019). The accuracy also increased to 0.858, a 5.6% improvement (McNemar test, *p* = 0.035), and specificity and sensitivity reached 0.840 and 0.835, respectively—improving by 2.7% and 2.4%, both of which were statistically significant.

By combining both pre- and post-chemotherapy images, our model significantly enhanced the comprehensiveness and accuracy of NACT efficacy prediction for breast cancer. This cross-temporal image fusion strategy plays a critical role in boosting prediction performance by effectively integrating key biomarker changes before and after treatment. Consequently, this approach provides a more precise assessment of treatment response, which is valuable for clinical decision-making.

### Ablation study and module effectiveness

**Figure 6:**
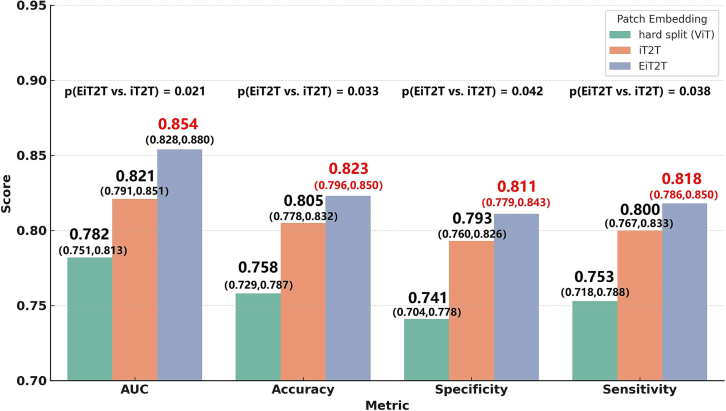
Ablation study of the EiT2T module with 95% CI and *p*-values Bars represent the mean ± 95% CI across the 5-fold cross-validation. Statistical significance for AUC was assessed using the DeLong test, while accuracy, specificity, and sensitivity were assessed using paired t-tests. The EiT2T module demonstrated significantly higher AUC, accuracy, specificity, and sensitivity compared with both the hard-split ViT and iT2T baselines (*p* < 0.05).

We conducted a series of ablation experiments to comprehensively evaluate the key improvements made to the DIST model and to verify the significant contributions of these innovations to the model’s performance. All ablation experiment results were based on test set data. The first set of experiments focused on the EiT2T module, for which we designed two comparison baselines: (1) a conventional ViT module using standard hard-tokenized patch embeddings, (2) the previously proposed iT2T module by Tong et al.,^29^ which employs two independent T2T modules to process images from different perspectives, enabling richer feature representations. To eliminate confounding factors, neither configuration incorporated positional or temporal embedding modules, and both used the CLS token as the global feature representation. The comparative results of these different patch embedding strategies are summarized in Figure 6. We observed that the enhanced T2T embedding method based on the EiT2T module outperformed the alternative configurations across all evaluation metrics. Specifically, compared to the iT2T baseline, the AUC improved from 0.821 to 0.854, and accuracy increased from 0.805 to 0.823. Both specificity and sensitivity improved by approximately 1.8 percentage points. This performance difference was statistically significant according to the DeLong test (*p* = 0.021), indicating that the EiT2T module effectively enhances recognition accuracy by introducing multi-scale feature extraction and fusing local-global attention mechanisms on top of the iT2T structure.

**Figure 7:**
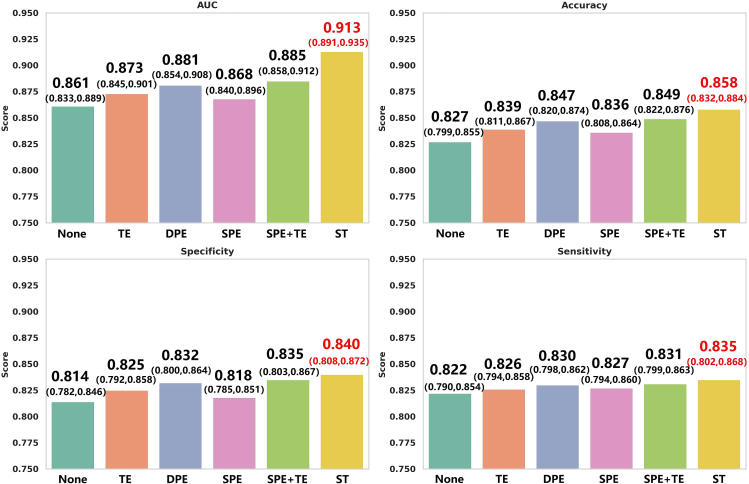
Ablation study of the ST module with 95% CI Bars represent the mean ± 95% CI across the 5-fold cross-validation. Statistical significance for AUC was assessed using the DeLong test, while accuracy, specificity, and sensitivity were assessed using paired t-tests. The full ST module produced the strongest overall performance, outperforming all intermediate variants and the baseline configuration.

Next, we conducted ablation studies on the ST module while keeping other conditions constant, including the consistent use of the EiT2T and AFFC modules. We designed six experimental configurations: (1) Baseline model without the ST module; (2) Model containing only the Temporal Embedding (TE) module; (3) Model containing only the Dynamic Position Embedding (DPE) module; (4) Model containing only the SPE module proposed by Tong et al.^29^; (5) Model containing both the SPE module proposed by Tong et al.^29^ and the TE module; (6) Model containing the complete ST module without any removals. The ablation results are presented in Figure 7, where each experimental model exhibited distinct performance differences. The baseline model, which did not use the ST module, showed the lowest performance, highlighting the importance of the ST module for the overall model effectiveness. In models containing only position embedding or only temporal embedding, we observed certain performance improvements, each with a specific focus. Specifically, position embedding helps handle changes in tumor morphology and size at relatively fixed positions, while temporal embedding is more sensitive to capturing the temporal dynamics of tumor morphology and size changes. In the DPE module, performance significantly improved compared to the SPE module, indicating that dynamically generated position embeddings better adapt to spatial variations in different images. Additionally, when position embedding and the TE module were used together, model performance further improved, suggesting that their combination is complementary and enhances the ability to analyze complex spatial and temporal dynamics. Finally, the ST module, which integrates DPE and TE modules, showed the best performance, outperforming other model configurations in all evaluation metrics, including AUC, Accuracy, Specificity, and Sensitivity. This confirms that the design of the ST module maximizes the use of spatiotemporal information, significantly enhancing the model’s recognition ability and accuracy. Overall, the results demonstrate the strong advantage of the ST module in handling complex medical image data, especially in tasks requiring the analysis of spatial and temporal dynamic changes.

**Figure 8:**
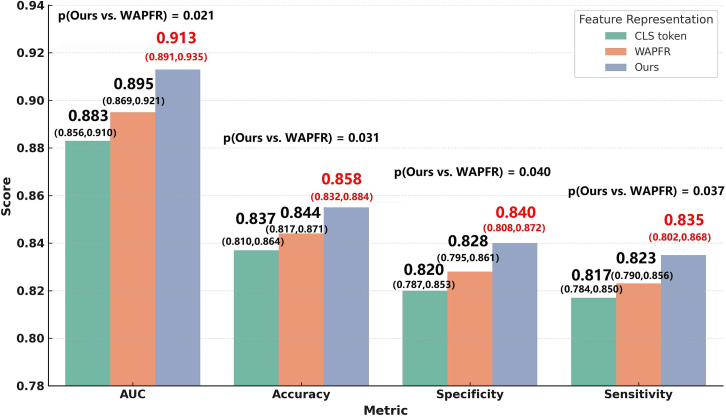
Ablation study of the AFFC module with 95% CI and *p*-values Bars represent the mean ± 95% CI across the 5-fold cross-validation. Statistical significance for AUC was evaluated using the DeLong test, while accuracy, specificity, and sensitivity were evaluated using paired t-tests. The AFFC module substantially outperformed both CLS and WAPFR across all metrics (*p* < 0.05), highlighting its effectiveness in capturing temporal changes between pre- and post-treatment imaging.

Finally, we conducted an ablation study on the AFFC module by comparing three different feature representation strategies: the conventional CLS token, the WARFR module proposed by Tong et al.,^29^ and our proposed AFFC module. All experiments were conducted using the same backbone network (EiT2T + ST) to ensure a fair comparison. As shown in Figure 8, the model using the CLS token achieved the lowest performance across all evaluation metrics, indicating its limited ability to effectively capture temporal and semantic information in complex medical images. The WARFR module, which incorporates an additional feature fusion mechanism, led to moderate improvements over the CLS baseline, with increases of 1.2%, 0.7%, 0.8%, and 0.6% in AUC, accuracy, specificity, and sensitivity, respectively. However, these improvements remained limited when compared to our AFFC module. Specifically, the AFFC module achieved an AUC of 0.913, significantly outperforming WARFR (AUC = 0.895, *p* = 0.021). The accuracy improved to 0.858, compared to 0.844 for WARFR (*p* = 0.031). In addition, the AFFC module yielded a 1.2% improvement in both specificity and sensitivity, with *p*-values of 0.040 and 0.037, respectively. These differences were statistically validated using the DeLong test and independent samples t-tests. These findings further confirm the advantage of the AFFC module in modeling features across multiple time points. By leveraging an adaptive weighting mechanism, the AFFC module enhances the temporal contextual representation, thereby improving the model’s ability to perceive and classify lesion changes before and after treatment.

## Discussion

In this study, we thoroughly explored the feasibility of customizing a model specifically for predicting the efficacy of NACT. Through extensive and rigorous experiments, we demonstrated that models incorporating meticulously designed embedding modules outperform traditional ViT models, achieving more competitive and significantly superior performance. Specifically, we introduced the DIST model, an NACT efficacy prediction model tailored for breast MRI images. This model integrates embedding modules such as EiT2T, ST, and AFFC, which meticulously extract image features to maximize predictive performance.

In detail, the EiT2T module enhances the traditional T2T module by further improving the capture of detailed features such as image edges and textures. This enhancement allows the model to more accurately identify subtle changes in breast MRI images, thereby improving the representation of pathological features. The ST module effectively fuses spatial and temporal information, increasing the model’s sensitivity to pathological changes. By capturing the evolving characteristics of tumors at different time points, the model can more accurately predict chemotherapy outcomes. The AFFC module enhances the model’s discriminative ability by adaptively integrating features from different time points. This module dynamically adjusts the weights of features at each time point, fully utilizing multi-temporal information and enhancing the model’s adaptability to heterogeneous data.

### Limitations of the study

Despite the notable performance improvements achieved by our proposed method in predicting the efficacy of NACT, this study has several limitations. First, the research is based on a retrospective design, and the model relies on ultrasound images acquired at two time points—before and after treatment. Consequently, it primarily serves to classify treatment response or pathological remission, including assisting in decisions on whether to continue subsequent chemotherapy cycles, which may limit its applicability in real-time clinical decision-making. Future studies should explore predictive models based solely on pre-treatment data to develop tools with greater prospective value and decision-support potential. Second, the current model does not incorporate clinicopathological variables such as patient age, tumor staging, hormone receptor status, and molecular subtype. These factors are known to be strongly associated with treatment outcomes and could further enhance the model’s predictive accuracy and clinical utility if integrated into the training and validation processes. In addition, the relatively limited sample size may restrict the generalizability of the model. Further validation using larger, multi-center datasets is warranted to confirm its robustness and applicability across diverse patient populations.

Lastly, the applicability of the proposed DIST model to other cancer types, such as lung or gastric cancer, remains to be investigated. Given the differences in imaging characteristics and treatment response mechanisms across malignancies, evaluating the model’s transferability to various clinical contexts will be essential for assessing its broader generalizability.

Regarding the clinical endpoint, we acknowledge that pathological complete response (pCR) is widely recognized as the gold standard for evaluating NACT efficacy. However, in this study, we adopted the imaging-based clinical response defined by RECIST 1.1 (CR/PR vs. SD/PD) as the prediction endpoint. This decision was primarily due to the retrospective nature of our dataset, in which pCR information was not uniformly available for all patients. Moreover, RECIST-based response evaluation is a clinically accepted and routinely applied criterion for monitoring therapeutic efficacy during NACT, particularly when assessing longitudinal imaging changes. We also recognize that discrepancies between pCR and RECIST assessments may exist. While pCR provides pathological confirmation of tumor eradication, RECIST reflects dynamic morphological changes observable through imaging. Such discordance may arise from differences in evaluation timing and measurement criteria. Nevertheless, RECIST remains valuable for early and non-invasive assessment, offering practical clinical relevance in real-world treatment monitoring and model generalization across imaging-based studies.

## Resource availability

### Lead contact

• Further information and requests for resources and reagents should be directed to and will be fulfilled by the lead contact, Yourong Chen (chenyr@zjsru.edu.cn).

### Materials availability

• This study did not generate new unique reagents.

### Data and code availability

• This study analyzed existing, publicly available data from the I-SPY2 TRIAL database (https://www.ispytrials.org/). Additional institutional DCE-MRI data are not publicly available due to privacy regulations, but can be shared upon reasonable request.
• All original code for the Dual-Input spatiotemporal transformer (DIST) model has been deposited at GitHub (https://github.com/DONGGUANLIANG/-main-module-of-DIST) and is publicly available as of the date of publication.
• Any additional information required to reanalyze the data reported in this article is available from the lead contact upon request.

## Acknowledgments

This work was supported by Applied Basic Research Program Project of Zhejiang Province of China under Grant No. 2025C02G1991657 and Key Laboratory of Artificial Organs and Computational Medicine of Zhejiang Province under Grant No. SZD2025B016.

## Author contributions

G.D. curated data, developed software, and drafted the article. Z.W. led conceptualization, methodology, and edited the article. H.S. and H.C. conducted analysis and validation. L.L. reviewed and edited the article. Y.C. managed funding and project oversight and approved the final article. All authors reviewed the article.

## Declaration of interests

The authors declare no competing interests.

## STAR★Methods

### Key resources table

**Table:** 

REAGENT or RESOURCE	SOURCE	IDENTIFIER
**Deposited data**		

Breast DCE-MRI images	The Cancer Imaging Archive	https://www.cancerimagingarchive.net/collection/ispy2/
Code for NACT prediction	This paper	https://github.com/DONGGUANLIANG/-main-module-of-DIST

**Software and algorithms**		

PyTorch	PyTorch Foundation	https://pytorch.org/
Python	Python Software Foundation	https://www.python.org

### Experimental model and study participant details

This study was approved by Medical and Life Science Ethics Committee of Zhejiang Shuren University (Approval No. 202501086). All patients provided informed consent for translational studies.

### Method details

#### Network architecture

We designed and implemented a model called the DIST, specifically developed for predicting NACT efficacy. This model employs three core encoding modules, each adapted and optimized based on the foundational ViT architecture: 1) Enhanced isolated Tokens-to-Token Embedding Module (EiT2T): This module provides an innovative image processing approach that combines multi-scale feature extraction with a local-global attention mechanism. By effectively capturing image features across different scales and balancing detailed and global information, EiT2T significantly enhances the model’s ability to recognize complex lesions. 2) Spatio-Temporal Embedding Module (ST): This module integrates dynamic positional embeddings with temporal embeddings, merging spatial and temporal information into a unified embedding strategy. By dynamically generating unique embedding vectors for each image, the module enhances the model's ability to detect spatial and temporal variations, accurately reflecting changes across both dimensions. 3) Adaptive Feature Fusion and Classification Embedding Module (AFFC): This module adaptively fuses features from different time points and calculates feature differences, improving the model's discriminative capability. By focusing on the features that contribute most to the classification task, the model effectively captures changes in lesion areas before and after treatment.

The overall structure of the DIST model is illustrated in Figure S1. The model receives a pair of medical images captured at different time points (pre- and post-chemotherapy). Each image is initially divided into multiple small patches, which are preliminarily encoded by the EiT2T module. This stage utilizes multi-scale feature extraction and a local-global attention mechanism to preserve the local attributes of the image while efficiently capturing complex lesion information across various scales. Next, the encoded image patches are further enhanced by the ST module. This module introduces combined dynamic positional embeddings and temporal embeddings, directly integrating spatial and temporal information into each encoded token, thus enabling a deeper understanding of spatio-temporal correlations. This enhancement allows the model to be more sensitive to spatio-temporal changes in lesion areas when processing pre- and post-chemotherapy images. Following feature enhancement, the AFFC module takes over. The AFFC module employs an adaptive weighting mechanism to merge features from the pre- and post-chemotherapy images, focusing on those most pertinent to the classification task. It also calculates feature differences to directly model the temporal changes in lesions. Finally, the combined and differentiated features contribute to the final classification decision.

#### Implementation detail

We employed 5-fold cross-validation in our experiments and reported the average performance across all five folds for key evaluation metrics, including AUC, accuracy, sensitivity, and specificity. To enhance the robustness and generalizability of the results, 95% confidence intervals were estimated using the formula: mean ± 1.96 × standard error. Specifically, the model was trained using the cross-entropy loss function and the AdamW optimizer (β1 = 0.85 and β2 = 0.998), with a cosine annealing learning rate schedule. The initial learning rate was set to 0.0001, and the weight decay was set to 0.02. To prevent overfitting, an early stopping strategy was adopted, with model selection based on the minimum validation loss. For a fair comparison, the same early stopping strategy was applied to all models, including our proposed method and all baselines, instead of using a fixed number of training epochs. The final model used for testing was selected based on its best performance on the validation set.

All models were designed with dual input images (pre-chemotherapy and post-first-cycle chemotherapy imaging) as illustrated in Figure S2. Each neural network input consisted of combined data from six sequences (S0–S5), resulting in an input channel count of six. This study focused on efficacy prediction based on clinical efficacy evaluations. According to the efficacy criteria, CR and PR indicate effective chemotherapy, while SD and PD indicate ineffective chemotherapy; therefore, the model output was two-dimensional. All images were resized to 224 × 224 pixels. All experiments were conducted using the PyTorch framework and were performed on an NVIDIA A100 GPU for model training and testing.

#### Evaluation metrics

The performance metrics used to evaluate the efficacy of NACT in this study include the Area Under the Receiver Operating Characteristic Curve (AUC), Accuracy, Sensitivity, and Specificity. These metrics provide quantitative measures to assess the effectiveness of the model in predicting the outcomes of NACT.

AUC is a statistical metric for evaluating the classification performance of a model, representing the model’s ability to distinguish between positive and negative cases. The AUC ranges from 0 to 1, where an AUC of 1 represents a perfect classifier, and an AUC of 0.5 indicates a model that performs no better than random guessing. The AUC is calculated as follows:

$$AUC=\int_{0}^{1}TPR\left(\theta \right)dFPR$$

where *TPR*(*θ*) is the True Positive Rate at threshold *θ*, and *FPR* is the False Positive Rate.

Accuracy represents the proportion of correct predictions made by the model and is one of the most straightforward performance metrics. It is calculated as follows:

$$Accuracy=\frac{TP+TN}{TP+FP+FN+TN}$$

where *TP* is the number of true positives, *TN* is the number of true negatives, *FP* is the number of false positives, and *FN* is the number of false negatives.

Sensitivity reflects the model’s ability to correctly identify positive cases. It is calculated as follows:

$$Sensitivity=\frac{TP}{TP+FN}$$

Specificity measures the model’s ability to correctly identify negative cases. It is calculated as follows:

$$Specificity=\frac{TN}{TN+FP}$$

#### Enhanced isolated tokens-to-token embedding module

In our model, inspired by the progressive tokenization strategy of the T2T image encoding module proposed by Li et al.,^30^ and the integration of dual T2T modules in the DiT model by Tong et al.^29^ for predicting NACT efficacy in breast ultrasound images, we have improved upon the conventional T2T module and developed an EiT2T Module. Unlike the conventional ViT approach, which tokenizes the image by directly dividing it into patches, our EiT2T module combines multi-scale feature extraction with a local-global attention mechanism. This approach not only retains important edge, texture, and other structural information more effectively, but also significantly enhances the model's ability to capture complex lesion features within pre- and post-treatment images, which is critical for the analysis of complex medical images. The module design includes two branches with non-shared parameters, each processing pre- and post-NACT images independently, as shown in the upper part of Figure S3. This dual-branch setup accommodates variations in tumor characteristics across different treatment stages.

The EiT2T module consists of three main stages: multi-scale feature extraction, local-global attention fusion, and soft split, as illustrated in the lower part of Figure S3. In the first two stages, we introduce multi-scale convolutions and a local-global attention mechanism. These innovations allow the module to capture image features at multiple scales while balancing fine details with global context, thereby significantly improving the model's ability to recognize complex lesion patterns. This technique ensures the preservation of high-resolution image details along with the critical features of lesions. During the soft split stage, the processed feature maps are further refined to optimize the token sequence, thereby ensuring that the inputs are of high quality for subsequent deep feature extraction and analysis. By employing overlapping patch division, the model effectively avoids the loss of structural information, retaining essential spatial relationships and features. This enhanced tokenization strategy is crucial for providing high-resolution, structurally-rich inputs that form a strong foundation for downstream analysis, ultimately improving the model's accuracy in identifying and differentiating tumor characteristics across treatment stages.

##### Multi-Scale feature extraction

Lesion regions in medical images often vary in size and shape, making multi-scale feature extraction essential for accurately identifying these areas. To this end, we perform multi-scale feature extraction on the token sequences at the input stage. Suppose the input of the *i-th* EiT2T block consists of token sequences $T_{before}^{i}$ ∈$R^{l_{i}\times c}$ and $T_{after}^{i}$ ∈$R^{l_{i}\times c}$ from before and after chemotherapy, where R denotes the real matrix space with *l*_*i*_ rows and *c* columns, representing the token length and embedding dimension, respectively.

We first process these token sequences through two isolated Transformers:

$$F_{before}^{i}={Transformer}_{before}\left(\;T_{before}^{i}\right)$$

$$F_{after}^{i}={Transformer}_{after}\left(\;T_{after}^{i}\right)$$

where each Transformer comprises multi-head self-attention and feed-forward networks. *Transformer*_*before*_ and *Transformer*_*after*_ refer to the Transformers handling the pre- and post-NACT token sequences, respectively. The outputs $F_{before}^{i}$ and $F_{after}^{i}$ represent the feature representations of pre- and post-chemotherapy images processed by the *i-th* EiT2T block.

Next, we reshape these feature representations into feature maps with spatial dimensions:

$$I_{before}^{i}=Reshape\left(F_{before}^{i}\right)$$

$$I_{after}^{i}=Reshape\left(F_{after}^{i}\right)$$

where *Reshape* denotes the reshaping operation. $I_{before}^{i}$ and $I_{after}^{i}$ represent the pre- and post-treatment feature maps, with dimensions converted from $R^{l_{i}\times c}$ to $R^{h\times w\times c}$, h and w are the height and width that satisfy *l*_*i*_=h×w.

On the reshaped feature maps, we perform multi-scale feature extraction. By applying convolutions with kernels of different sizes (e.g., k1×k1, k2×k2, ……, kn×kn), we obtain feature representations at different scales:

$$I_{before}^{i,s_{j}}={Conv}_{k_{j}}\left(\;I_{before}^{i}\right)$$

$$I_{after}^{i,s_{j}}={Conv}_{k_{j}}\left(\;I_{after}^{i}\right)$$

where $I_{before}^{i,s_{j}}$ and $I_{after}^{i,s_{j}}$ represent the pre- and post-treatment feature maps at the *j-th* scale *s*_*j*_. The index *j* denotes the scale, with each scale *s*_*j*_ corresponding to a convolution with kernel size *k*_*j*_×*k*_*j*_. The convolution operation ${Conv}_{k_{j}}$ applies a kernel of size *k*_*j*_ to the feature maps, capturing features at various scales.

##### Local-Global attention fusion

At various scales of the feature maps, we further employ local and global attention mechanisms to effectively capture both detailed and overall information. The local attention mechanism focuses on extracting detailed information within local regions. For each scale of the feature map, we define a local window (with a window size of ω = 7 × 7, which can be adaptively adjusted according to the input resolution.) and compute self-attention within the window:

$$F_{before-l}^{i,s_{j}}=LocalAttn\left(\;I_{before}^{i,s_{j}}\right)$$

$$F_{after-l}^{i,s_{j}}=LocalAttn\left(\;I_{after}^{i,s_{j}}\right)$$

where $F_{before-l}^{i,s_{j}}$ and $F_{after-l}^{i,s_{j}}$ represent the pre- and post-chemotherapy features at scale *s*_*j*_ after processing with the local attention mechanism, denoted by *LocalAttn*. The local self-attention is calculated as:

$$LoaclAttn\left(Q,K,V\right)=\text{Softmax}\left(\frac{QK^{T}}{\sqrt{d_{k}}}\right)V$$

where *Q*, *K*, and *V* are the query, key, and value matrices derived from the input features $I^{i,s_{j}}$ through linear transformations, and *d*_*k*_ is the dimension of the key. By computing attention only within the local window, we reduce computational complexity while retaining fine-grained information.

The global attention mechanism, on the other hand, captures broader context across the entire feature map by computing self-attention over all regions:

$$F_{before-g}^{i,s_{j}}=GlobalAttn\left(\;I_{before}^{i,s_{j}}\right)$$

$$F_{after-g}^{i,s_{j}}=GlobalAttn\left(\;I_{after}^{i,s_{j}}\right)$$

where $F_{before-g}^{i,s_{j}}$ and $F_{after-g}^{i,s_{j}}$ are the pre- and post-chemotherapy features at scale *s*_*j*_ after applying the global attention mechanism, represented by *GlobalAttn*. The calculation of global self-attention is similar to that of local self-attention, but operates over the entire feature map:

$$GlobalAttn\left(Q,K,V\right)=\text{Softmax}\left(\frac{QK^{T}}{\sqrt{d_{k}}}\right)V$$

We then fuse the results of local and global attention by applying adaptive weighting to obtain a combined feature representation, Local attention is applied within non-overlapping windows to capture fine-grained local textures and edge details, whereas global attention operates over the entire feature map to model long-range dependencies. The outputs of the two attention branches are then fused through weighted summation, allowing the network to exploit both local and global contextual cues simultaneously while maintaining computational efficiency:

$$F_{before}^{i,s_{j}}=\alpha^{s_{j}}\cdot F_{before-l}^{i,s_{j}}+\beta^{s_{j}}\cdot F_{before-g}^{i,s_{j}}$$

$$F_{after}^{i,s_{j}}=\alpha^{s_{j}}\cdot F_{after-l}^{i,s_{j}}+\beta^{s_{j}}\cdot F_{after-g}^{i,s_{j}}$$

where $F_{before}^{i,s_{j}}$ and $F_{after}^{i,s_{j}}$ are the fused pre- and post-treatment features at scale *s*_*j*_ after combining local and global attention results. The weights $\alpha^{s_{j}}$ and $\beta^{s_{j}}$ control the contributions of local and global attention, respectively, and are learnable parameters that satisfy $\alpha^{s_{j}}$ +$\beta^{s_{j}}$=1.

After obtaining feature representations at different scales, we proceed to multi-scale feature fusion. The features from each scale are combined using learned weights $\omega^{s_{j}}$, which are obtained through a fully connected layer followed by a Softmax function to ensure they sum to 1:

$$F_{before}^{i,fused}=\sum_{j=1}^{n}\omega^{s_{j}}\cdot F_{before}^{i,s_{j}}$$

$$F_{after}^{i,fused}=\sum_{j=1}^{n}\omega^{s_{j}}\cdot F_{after}^{i,s_{j}}$$

where $F_{before}^{i,fused}$ and $F_{after}^{i,fused}$ represent the fused multi-scale features for the pre- and post-chemotherapy images, respectively. The weights $\omega^{s_{j}}$ are scale-specific coefficients learned adaptively through the model, ensuring that the model can prioritize the most informative scales based on the input data.

This method enables the model to dynamically modulate the significance attributed to various scales and attention types, thereby improving its capacity to discern both local nuances and overarching contexts.

##### Soft split

After fusing the reshaped multi-scale feature maps, we apply a soft split operation to the resulting feature maps. Soft split is an expansion operation that divides the feature maps into overlapping patches using a kernel of size *k* and a stride of *s*:

$$T_{before}^{i+1}=Unfold\left(F_{before}^{i,fused}\right)$$

$$T_{after}^{i+1}=Unfold\left(F_{after}^{i,fused}\right)$$

where $T_{before}^{i+1}$ and $T_{after}^{i+1}$ denote the token sequences for the pre- and post-chemotherapy images after soft split, and *Unfold* represents the soft split operation.

The benefit of soft splitting is that it creates overlapping patches, which helps preserve the structural information of the images and prevents the loss of important features. By retaining the continuity and spatial relationships between patches, this approach ensures that critical details are not missed during the tokenization process.

The resulting token sequences $T_{before}^{i+1}$ and $T_{after}^{i+1}$ serve as inputs to the next EiT2T block in the model pipeline. This overlap between patches enhances the representation of fine-grained features, providing richer contextual information for subsequent processing, ultimately contributing to improved accuracy in capturing subtle changes and predicting NACT efficacy.

#### Spatio-Temporal embedding module

When processing medical image data, although self-attention mechanisms can effectively capture global features, they often overlook the inherent spatio-temporal relationships between image tokens. In particular, when dealing with time-series medical images, traditional self-attention mechanisms fail to fully utilize the temporal information between images, limiting their potential in dynamic medical imaging analysis. To address this issue, we propose the ST module, which aims to optimize the performance of self-attention mechanisms when handling medical images involving temporal variations.

Although traditional transformer models share the same framework for positional and temporal embeddings, the processing of these two types of information remains relatively independent, failing to fully integrate spatial and temporal information. To address this issue, we introduce dynamic positional embeddings and combine them with temporal embeddings. This approach significantly simplifies the model's complexity, enhances processing efficiency, and improves information integration capabilities. It not only strengthens the model's ability to capture pathological changes in images but also increases the accuracy and efficiency of analysis, thereby enhancing the model's understanding and utilization of tumor features as they change over time.

##### Dynamic position embedding

Traditional positional embeddings utilize fixed positional matrices shared across all images, as demonstrated by Tong et al.^29^ in their Shared Position Embedding (SPE) module. Such static embeddings fail to account for variations between individual images, particularly in multi-time-point datasets where changes in imaging angles and tumor morphology can alter spatial structures. To overcome this limitation, we introduce Dynamic Position Embedding (DPE), which dynamically generates unique embedding vectors for each image. This method allows for adaptive adjustments tailored to the specific characteristics of each image, effectively capturing differences between individuals.

Consider the *n-th* sample in the dataset, with its pre-chemotherapy image denoted as $X_{before}^{n}$ and post-chemotherapy image as $X_{after}^{n}$. After processing through the modules in EiT2T, these images are transformed into the corresponding token sequences $T_{before-E}^{n}$ and $T_{after-E}^{n}$. Using the dynamic position embedding function *E*_*pos*_, we generate position embedding matrices for the pre- and post-treatment images as follows:

$${DPE}_{before}^{n}=E_{pos}\left(T_{before-E}^{n}\right)$$

$$D{PE}_{after}^{n}=E_{pos}\left(T_{after-E}^{n}\right)$$

where ${DPE}_{before}^{n}$ and $D{PE}_{after}^{n}$ represent the dynamic position embedding matrices for the pre- and post-chemotherapy images of the $n-th$ sample, respectively. The function *E*_*pos*_ is a dynamic position embedding function generated through a convolutional neural network, unlike traditional sinusoidal or cosine embeddings. This convolutional approach allows *E*_*pos*_ to dynamically adjust according to the local features of each image.

By generating separate dynamic position embedding matrices for the pre- and post-treatment images, the model can better capture spatial differences between these two image sets. The formula for calculating the dynamic position embedding function is as follows:

$$E_{pos}\left(T\right)=W_{pos}\cdot f\left(T\right)+b_{pos}$$

where *W*_*pos*_ is a learnable weight matrix, *f*(*T*) represents the convolutional feature extraction operation applied to the input token sequences $T_{before-E}^{n}$ and $T_{after-E}^{n}$, and *b*_*pos*_ is a bias vector used to adjust the generated dynamic position embeddings.

In our implementation, the dynamic position embedding function is realized using a lightweight convolutional neural network consisting of three convolutional layers with kernel sizes of 3×3, 3×3, and 1×1, respectively. Each convolutional layer is followed by Batch Normalization and a ReLU activation to enhance nonlinearity and stabilize feature learning. The final 1×1 convolution is employed to project the extracted spatial features into the embedding dimension, ensuring compatibility with the transformer input tokens.

We adopted this CNN-based approach rather than traditional sinusoidal or cosine positional encodings because convolutional layers can dynamically adapt to the local texture and spatial variations of each image, thereby producing position embeddings that are data-dependent and sample-specific. This enables the model to more accurately capture morphological differences across patients and between pre- and post-chemotherapy images, which is particularly beneficial in multi-time-point medical imaging scenarios.

##### Spatio-Temporal fusion embedding

Before and after chemotherapy, the morphology and position of tumors change, and these changes are interrelated in both spatial and temporal dimensions. To better capture this interrelation, we combine dynamic position embeddings with temporal embeddings, enhancing the model's understanding of tumor changes.

The temporal embedding design is based on sine and cosine functions, which are well-suited for capturing periodic characteristics in time series data. Let t=0 represent the pre-chemotherapy image and t=1 represent the post-chemotherapy image. The temporal embedding *TE*_*t*_ is computed as follows:

$${TE}_{t}\left[2j\right]=\sin\left(\frac{t}{10000^{\frac{2j}{d}}}\right)$$

$${TE}_{t}\left[2j+1\right]=\cos\left(\frac{t}{10000^{\frac{2j}{d}}}\right)$$

where *TE*_*t*_ denotes the temporal embedding vector for time *t* (with *t*=0 indicating pre-chemotherapy and *t*=1 indicating post-chemotherapy). The index *j* represents the position in the embedding dimension, alternating between sine and cosine values, and *d* is the total dimension of the embedding vector.

By combining dynamic position embeddings with temporal embeddings, we create mixed spatio-temporal embeddings for the pre- and post-chemotherapy images as follows:

$${ST}_{before}^{n,t}={DPE}_{before}^{n}+{TE}_{0}$$

$${ST}_{after}^{n,t}={DPE}_{after}^{n}+{TE}_{1}$$

where ${ST}_{before}^{n,t}$ and ${ST}_{after}^{n,t}$ represent the spatio-temporal embeddings for the pre- and post-chemotherapy images, respectively. This fusion allows the model to simultaneously capture spatial and temporal changes in the images, enabling it to better adapt to morphological differences in the tumor before and after treatment.

During model training and inference, the token sequences *T* generated from the previous module are combined with the spatio-temporal embedding matrices:

$$T_{before-st}^{n}=T_{before-E}^{n}+{ST}_{before}^{n,t}$$

$$T_{after-st}^{n}=T_{after-E}^{n}+{ST}_{after}^{n,t}$$

where $T_{before-st}^{n}$ and $T_{after-st}^{n}$ represent the token sequences for pre- and post-chemotherapy images after incorporating spatio-temporal embeddings.

In this module, the model processes the pre- and post-chemotherapy image tokens separately, combining dynamic position embeddings with temporal embeddings to capture spatial and temporal changes in the tumor. The pre-chemotherapy token sequence $T_{before-E}^{n}$ is added to its corresponding spatio-temporal embedding ${ST}_{before}^{n,t}$, and similarly, the post-chemotherapy token sequence $T_{after-E}^{n}$ is combined with ${ST}_{after}^{n,t}$ . This independent processing of each set of tokens with their respective spatio-temporal embeddings helps the model better understand the morphological changes in the tumor across the treatment timeline.

#### Adaptive feature fusion and classification embedding module

To effectively integrate features from pre- and post-chemotherapy images and enhance the model's ability to capture temporal changes in lesions, we propose the AFFC module. Unlike traditional classification methods that use a CLS token for decision-making, the AFFC module dynamically adjusts the feature weights from different time points, allowing the model to focus on the most contributive features for the classification task. By introducing feature differences, AFFC not only improves the model's discriminative capability but also prevents potential information loss and excessive reliance on global features associated with the CLS token. This enables the model to gain a deeper understanding of the changes in lesion areas before and after chemotherapy, thereby increasing prediction accuracy. The structure of this module is shown in Figure S4.

##### Feature extraction and adaptive fusion

After the preceding processing, assume we have obtained the embedded token sequences *T*_*before*-*st*_ and *T*_*after*-*st*_ for a given case, representing the pre- and post-chemotherapy images. These token sequences are then fed into a Transformer encoder with shared parameters to produce feature representations *F*_*before*-*st*_ and *F*_*after*-*st*_. To adaptively fuse the features extracted before and after treatment, we apply global average pooling (GAP) to each feature representation to obtain global feature vectors:

$$\bar{F_{before-st}}=GAP\left(F_{before-st}\right)$$

$$\bar{F_{after-st}}=GAP\left(F_{after-st}\right)$$

where *GAP*() denotes the global average pooling operation, and $\bar{F_{before-st}}$ and $\bar{F_{after-st}}$ represent the global feature vectors for the pre- and post-chemotherapy images, respectively.

These two global feature vectors are concatenated and passed through a fully connected (FC) layer, followed by a Softmax function, to generate fusion weights α and β:

$$\left[\alpha ,\beta \right]=Softmax\left(FC\left(\left[\bar{F_{before-st}};\bar{F_{after-st}}\right]\right)\right)$$

where [ ; ] denotes the concatenation operation, *FC* is the fully connected layer, and Softmax normalizes the result into a probability distribution to produce the weights α and β.

The adaptive weights are then used to perform a weighted fusion of the features:

$$F_{fused}=\alpha \cdot F_{before-st}+\beta \cdot F_{after-st}$$

where *F*_*fused*_ represents the fused feature representation.

This adaptive fusion enables the model to adjust the focus on pre- and post-chemotherapy features based on the characteristics of the input data, effectively leveraging the most informative features.

##### Classification decision

To capture the changes between pre- and post-chemotherapy features, we compute the feature difference Δ*F*, which directly reflects changes in the lesion areas before and after treatment and provides valuable discriminative information:

$$\Delta F=F_{after-st}-F_{before-st}$$

where Δ*F* represents the feature difference, offering the model significant insight for classification.

We then concatenate the fused feature *F*_*fused*_ and the feature difference Δ*F* along the feature dimension to form the final feature representation *F*_*final*_∈ $R^{N\times 2D}$:

$$F_{final}=\left[F_{fused};\Delta F\right]$$

To obtain a fixed-length vector for classification, we apply global average pooling to *F*_*final*_,resulting in a global feature vector $\bar{F_{final}}$:

$$\bar{F_{final}}=GAP\left(F_{final}\right)$$

where *F*_*final*_ contains both the overall information from the pre- and post-treatment features and specific information about their differences, and $\bar{F_{final}}$ represents the global feature vector after pooling.

Finally, the global feature vector is fed into a classifier, which consists of a fully connected layer followed by a Softmax function to output classification probabilities:

$$P=Softmax\left(FC\left(\bar{F_{final}}\right)\right)$$

where P∈ $R^{C}$ represents the classification probabilities for each category, with *C* being the number of classes. In this binary classification task, *C =* 2.

The predicted class label:

$$\hat{y}=\underset{c}{\mathrm{argmax}}P\left[c\right]$$

where $\hat{y}$ represents the final predicted label, *P*[*c*] is the probability of class *c*, and $\underset{c}{\mathrm{argmax}}$ selects the class with the highest probability.

### Quantification and statistical analysis

All statistical analyses were performed using Python (version 3.8). Model performance was quantified using receiver operating characteristic (ROC) analysis, and the area under the curve (AUC), accuracy, sensitivity, and specificity were computed across 5-fold cross-validation. AUC differences were assessed using the DeLong test, accuracy differences using the McNemar test, and specificity and sensitivity differences using paired t-tests. A p-value < 0.05 was considered statistically significant. No data were excluded, and all analyses were independently repeated to ensure reproducibility.

### Additional resources

This study did not create or use any additional websites or resources, and no clinical trial was registered.
