# Supplementary material for: Dual-input spatio-temporal transformer model: Predicting the efficacy of NACT in breast cancer based on DCE-MRI images
Source: iScience. 2025 Dec 13;29(1):114433. doi: 10.1016/j.isci.2025.114433 (PMC12796758; doi:10.1016/j.isci.2025.114433)
Supplement: Document S1. Figures S1–S4 [file mmc1.pdf]

**iScience, Volume *29***

## Supplemental information

# Dual-input spatio-temporal transformer model: Predicting the efficacy of NACT in breast cancer based on DCE-MRI images

**Hongbo Song, Guanliang Dong, Yourong Chen, Zhangquan Wang, Liyuan Liu, and Haidong Cui**

## Supplemental Materials

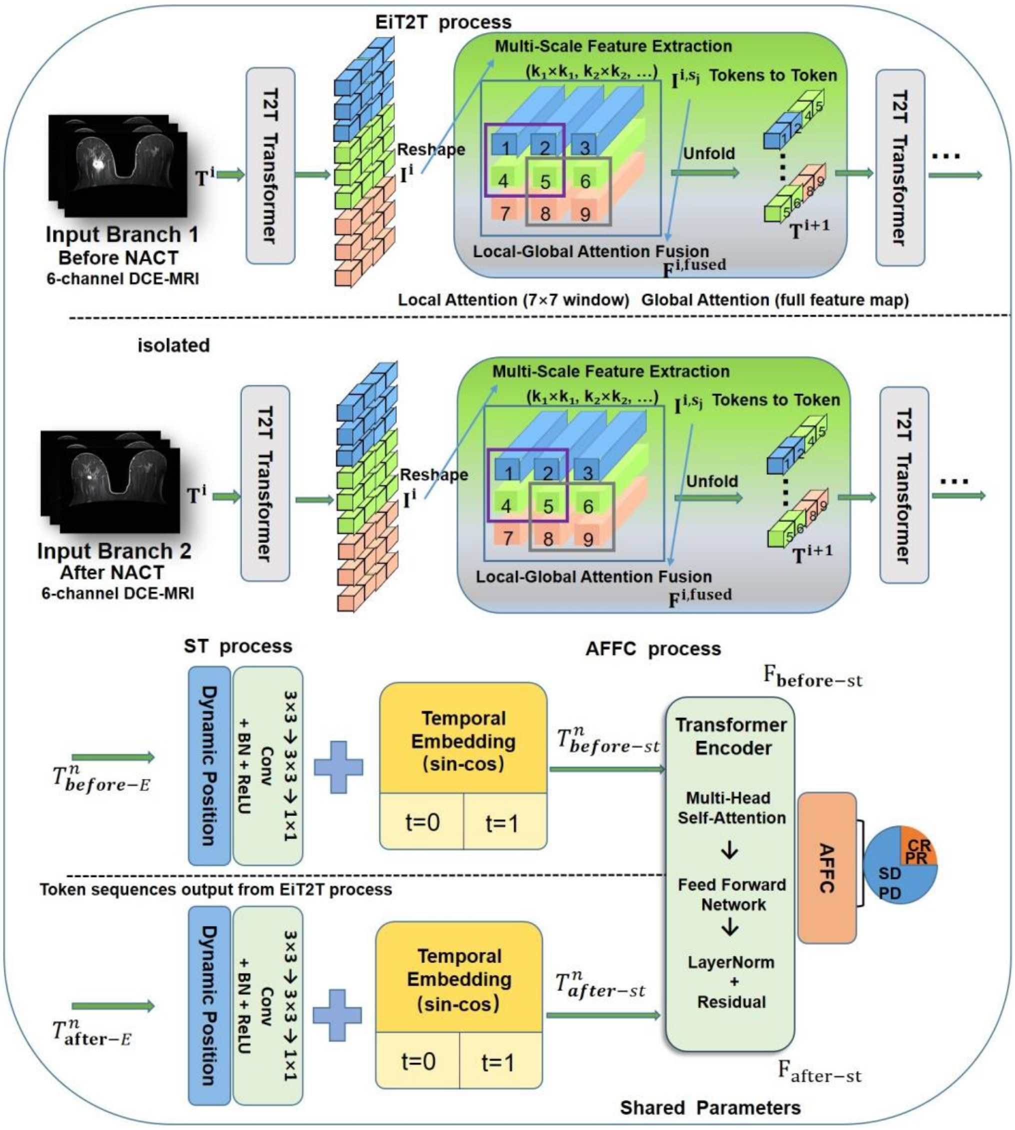


**Figure.S1 Structure of the DIST**

The specific structure of the AFFC module is illustrated in Figure.S4.

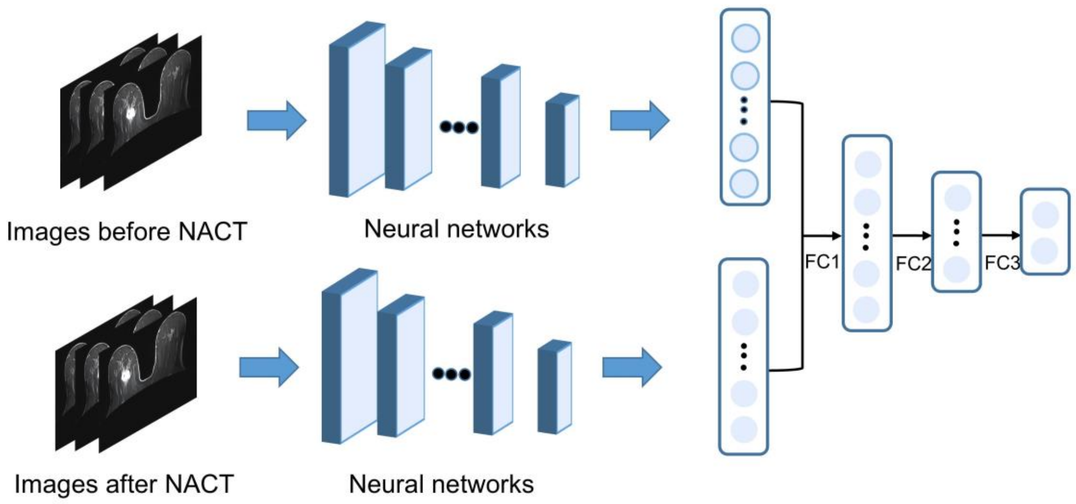


**Figure.S2 Structure of the Comparison Model**

The comparison model consists of two independent neural networks that process pre- and post-NACT images separately. The extracted feature vectors are concatenated and passed through fully connected layers for response prediction.

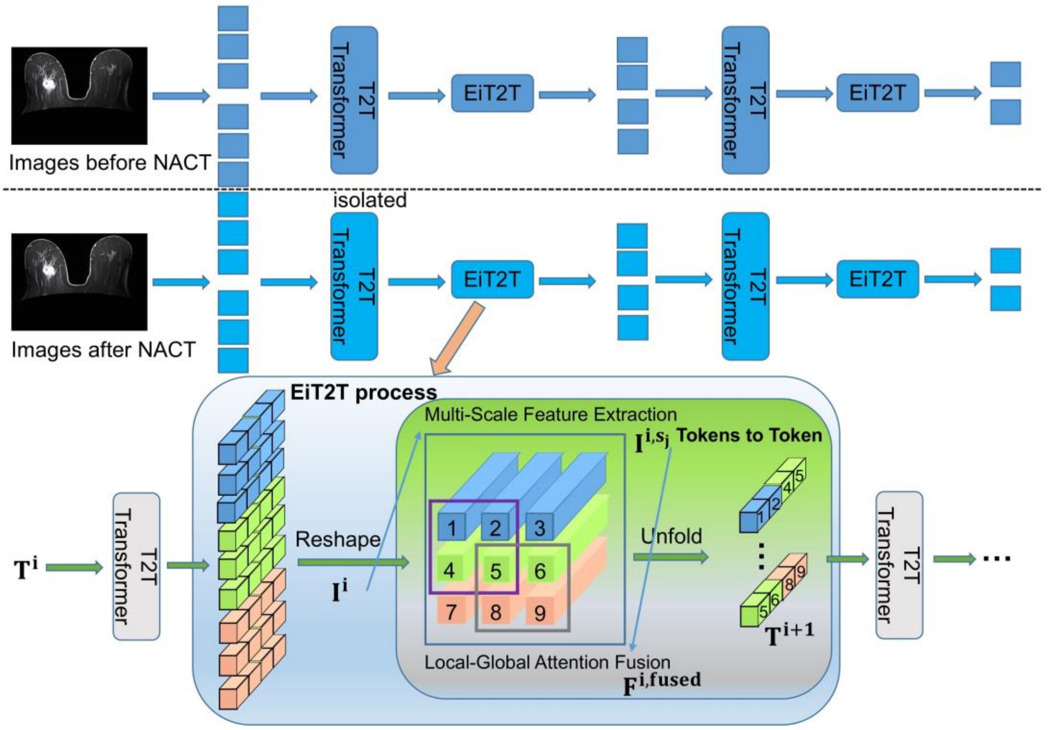


**Figure.S3 Structure of the EiT2T**

The EiT2T module enhances the conventional T2T transformer by introducing multi-scale feature extraction and a local–global attention fusion mechanism. Pre- and post-NACT images are processed independently through two non-shared transformer branches, enabling the model to effectively capture dynamic lesion features across treatment stages.

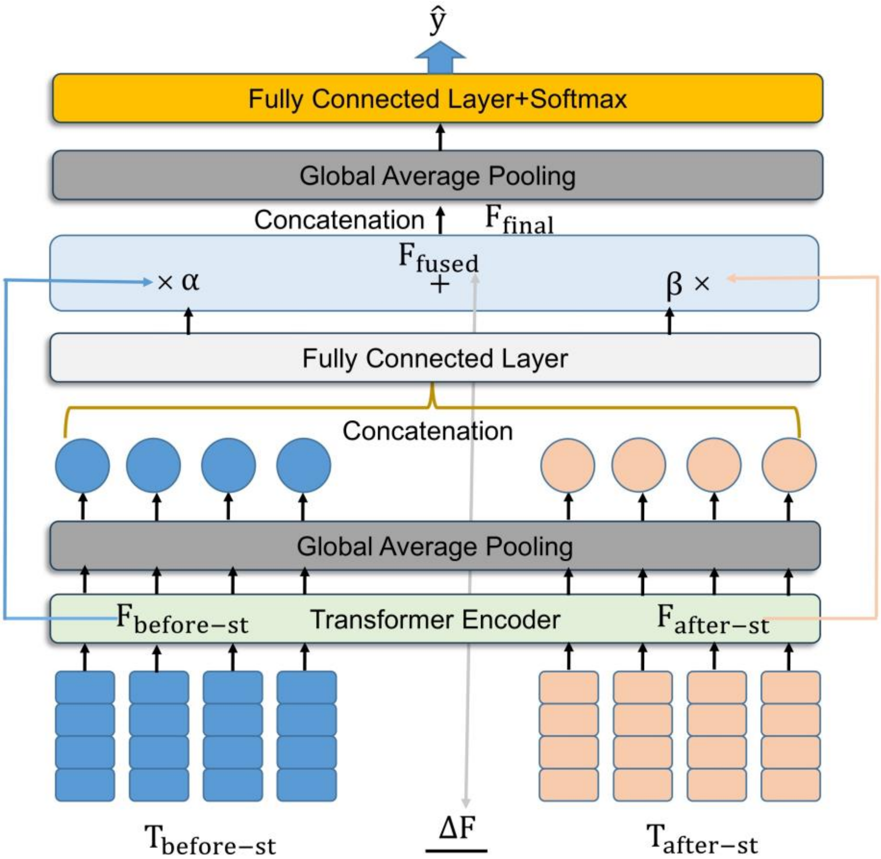


**Figure.S4 Structure of the AFFC**

The AFFC module adaptively fuses pre- and post-treatment features by weighting and combining their representations, emphasizing discriminative temporal changes for improved prediction accuracy.
